# Adjuvant Immune Enhancement of Subunit Vaccine Encoding pSCPI of *Streptococcus iniae* in Channel Catfish (*Ictalurus punctatus*)

**DOI:** 10.3390/ijms161226082

**Published:** 2015-11-25

**Authors:** Jie Jiang, Zonglin Zheng, Kaiyu Wang, Jun Wang, Yang He, Erlong Wang, Defang Chen, Ping Ouyang, Yi Geng, Xiaoli Huang

**Affiliations:** 1 Department of Basic Veterinary, College of Veterinary Medicine, Sichuan Agricultural University, Wenjiang District Huimin Road No. 211, Chengdu 611130, China; jiangjiejj2013@126.com (J.J.); wangjun1986616@gmail.com (J.W.); heyang@sicau.edu.cn (Y.H.); welsicau@126.com (E.W.); ouyang.ping@live.cn (P.O.); gengyisicau@126.com (Y.G.); 2 Department of Aquaculture, Rongchang Campus, Southwest University, Chongqing 402460, China; zhengzonglin@126.com; 3 Key Laboratory of Animal Disease and Human Health of Sichuan Province, Sichuan Agricultural University, Wenjiang District Huimin Road No. 211, Chengdu 611130, China; 4 Department of Aquaculture, Sichuan Agricultural University, Wenjiang District Huimin Road No. 211, Chengdu 611130, China; chendf_sicau@126.com (D.C.); hxldyq@126.com (X.H.)

**Keywords:** Channel catfish, *Streptococcus iniae*, subunit vaccine, pSCPI, adjuvant, immune effect

## Abstract

Channel catfish (*Ictalurus punctatus*) is an important agricultural fish that has been plagued by *Streptococcus iniae* (*S. iniae*) infections in recent years, some of them severe. C5a peptidase is an important virulent factor of *S. iniae*. In this study, the subunit vaccine containing the truncated part of C5a peptidase (pSCPI) was mixed with aluminum hydroxide gel (AH), propolis adjuvant (PA), and Freund’s Incomplete Adjuvant (FIA). The immunogenicity of the pSCPI was detected by Western-blot *in vitro*. The relative percent survival (RPS), lysozyme activity, antibody titers, and the expression of the related immune genes were monitored *in vivo* to evaluate the immune effects of the three different adjuvants. The results showed that pSCPI exerted moderate immune protection (RPS = 46.43%), whereas each of the three adjuvants improved the immune protection of pSCPI. The immunoprotection of pSCPI + AH, pSCPI + PA, and pSCPI + FIA was characterized by RPS values of 67.86%, 75.00% and, 85.71%, respectively. Further, each of the three different adjuvanted pSCPIs stimulated higher levels of lysozyme activity and antibody titers than the unadjuvanted pSCPI and/or PBS buffer. In addition, pSCPI + FIA and pSCPI + PA induced expression of the related immune genes under investigation, which was substantially higher than the levels stimulated by PBS. pSCPI + AH significantly stimulated the induction of MHC II β, CD4-L2, and IFN-γ, while it induced slightly higher production of TNF-α and even led to a decrease in the levels of IL-1β, MHC I α, and CD8 α. Therefore, we conclude that compared with the other two adjuvants, FIA combined with pSCPI is a more promising candidate adjuvant against *S. iniae* in channel catfish.

## 1. Introduction

Channel catfish (*Ictalurus punctatus*) is an important agricultural fish, especially in China where its yield was 150,000 tons in 2008 [1,2]. However, *Streptococcus iniae* (*S. iniae*) has seriously damaged fish farming in recent years [3,4]. *S. iniae*, a β-hemolytic Streptococcus species, has been an important fish pathogen with a broad host range, such as the hybrid striped bass (*Morone chrysops × M saxatilis*), tilapia (*Oreochromis* spp.), flounder (*Paralichthys dentatus*), rainbow trout (*Oncorhynchus mykiss*), channel catfish, and salmon (*Salmo salar*) [5,6,7,8]. Furthermore, this Gram-positive coccus also causes disease in human [6,9].

Fortunately, as green interventions to prevent *S. iniae* infection, vaccines are a good choice. In comparison with the inactivated and/or attenuated live vaccines, subunit vaccines composed of conserved proteins are safer and more serotype-independent [10]. However, poor immunogenicity has been the main constraint to subunit vaccine development. Thus, the use of adjuvants in subunit vaccines is exceedingly necessary and important. Adjuvants are a group of structurally heterogeneous compounds that can augment the immune response of a vaccine and modulate the intrinsic immunogenicity of an antigen, thus enhancing the protective immunity against the target diseases [11,12,13]. Fish vaccines frequently contain aluminum-based [12,14], oil-based [10,12,15,16], and propolis-containing [17,18,19] adjuvants. Aluminum adjuvants, such as aluminum phosphate and aluminum hydroxide, have been used for more than 70 years in humans, with proven safety [20,21]. Freund’s complete adjuvant (FCA) and Freund’s incomplete adjuvant (FIA) are common oil adjuvants with great immunoprotective efficacy. Propolis is a natural product, exhibiting immunomodulatory, anti-inflammatory, antimicrobial, antitumor, antioxidant, antiviral, antiparasitic, and anti-diabetic activities [22], due to which it is regarded as a potential adjuvant in veterinary vaccines.

C5a peptidase is a highly conserved, multifunctional surface protein. C5a peptidase of group A streptococci (GAS) has proven efficacy in humans and mice against GAS infections [23,24]. Likewise, C5a peptidase of group B streptococci (GBS) exerts immunogenicity in mice [24]. BLAST (tblastn) analysis indicated that the C5a peptidase obtained from *S. iniae* in the hybrid striped bass had equal degrees of similarity (37% identity, 55% positive) to the C5a peptidases of GAS and GBS [7]. We, therefore, speculated that C5a peptidase obtained from *S. iniae* might act as a valuable subunit vaccine for protection of fish against *S. iniae* infection.

However, since C5a peptidase is a macromolecule with a molecular weight of 123.4 kDa, its expression was difficult. Based on the results of previous investigations, in the present study, we truncated a part of C5a peptidase containing most of the B-cell epitopes (30–695 aa) and the conserved domains (designated pSCPI) to obtain high expression of recombinant protein.

The aim of this work was to investigate the immune enhancement of three different adjuvants against pSCPI. We used Western-blot analysis to confirm the presence of the pSCPI protein *in vitro*. Channel catfish were individually vaccinated to evaluate their immune protection *in vivo*. Subsequently, the lysozyme activity, serum bactericidal activity, antibody titer, and immune-related gene expression were used to assess the effect of the three adjuvants. All the experimental results indicated that FIA is a more promising candidate adjuvant combined with pSCPI against *S. iniae* in channel catfish.

## 2. Results

### 2.1. Western Blotting Analysis of the pSCPI Protein

**Figure 1:**
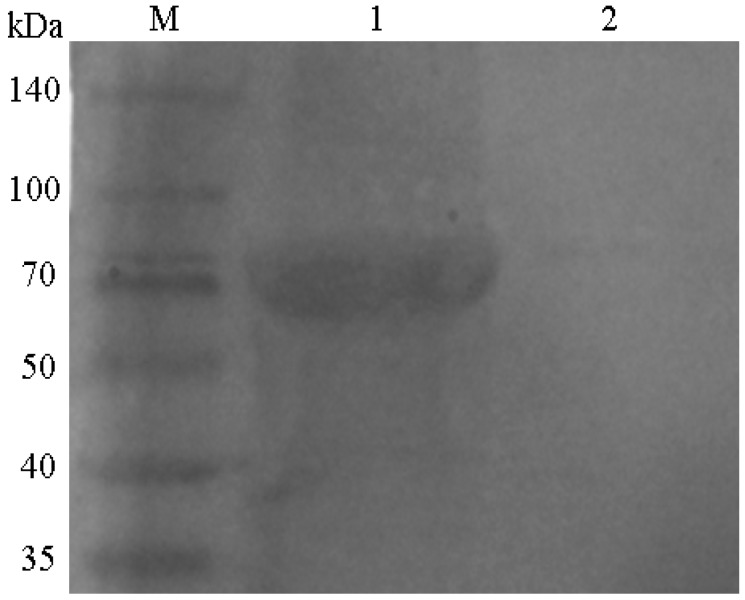
Western blot analysis of recombinant pSCPI. The proteins were separated by SDS-PAGE, transferred to a PVDF membrane, and blotted with anti-pSCPI or negative sera. M: Protein marker; 1: The recombinant pSCPI; 2: Negative control.

pSCPI proteins were quantified by SDS-PAGE and transferred to a PVDF membrane. Rabbit anti-pSCPI and negative sera were employed as primary antibodies for Western blotting. A specific band of approximately 87 KDa was determined in the rabbit anti-pSCPI experimental group, which was missing in the negative group (Figure 1, lanes 1 and 2). The results showed that pSCPI possessed immunogenicity.

### 2.2. Efficacy Against the S. iniae Strain DGX07

**Figure 2:**
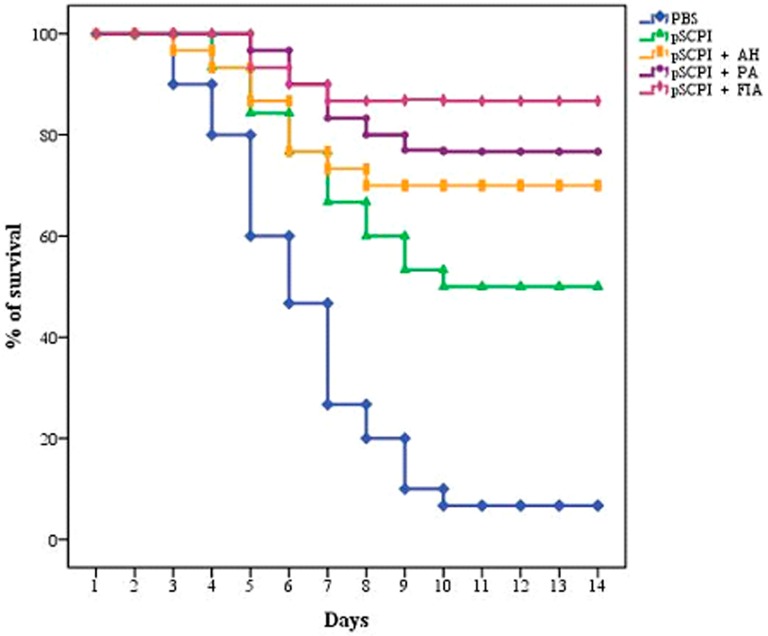
The survival rate of channel catfish after a lethal infection with the *S. iniae* strain DGX07. Channel catfish were vaccinated twice, at two-week intervals, with pSCPI + FIA, pSCPI + PA, pSCPI + AH, pSCPI, or PBS. Then, they were challenged i.p. through the use of 6 × 10^7^ CFU/fish of *S. iniae* strain DGX07 28 days after the last administration. Cumulative mortality rates were recorded daily for a 14-day period after the challenge. FIA: Freund’s Incomplete Adjuvant; PA: propolis adjuvant; AH: aluminum hydroxide gel.

Channel catfish were immunized with pSCPI + FIA, pSCPI + PA, pSCPI + AH, pSCPI, or PBS, followed by a challenge with 200 µL *S. iniae* strain DGX07 on the 28th day after the last administration. The quantity of RPS of pSCPI + FIA, pSCPI + PA, pSCPI + AH, and pSCPI was 85.71%, 75.00%, 67.86%, and 46.43%, respectively (Figure 2). The results of RPS demonstrated that: (1) pSCPI had a moderate capacity for immune protection; and (2) three different adjuvants improved the potential for immune protection, with the effect of FIA the most pronounced.

### 2.3. Lysozyme Activity

**Figure 3:**
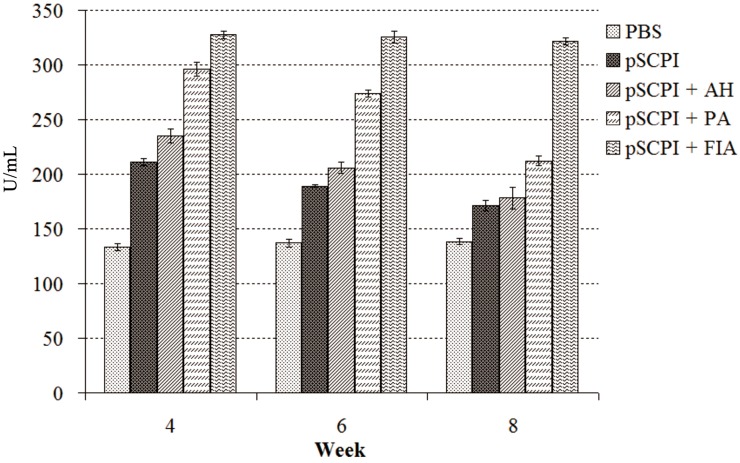
Serum lysozyme activity of vaccinated channel catfish. Channel catfish were vaccinated twice, 2 weeks apart, with PBS, pSCPI, pSCPI + AH, pSCPI + PA, and pSCPI + FIA. Sera from the three fish were collected and pooled at 4, 6, and 8 weeks post-vaccination.

The lysozyme activity was assessed by a turbidimetric assay 4, 6, and 8 weeks post-vaccination (Figure 3). On the whole, the four vaccine groups revealed higher levels of lysozyme activity than those of the control group. The three different adjuvanted pSCPI stimulated the activity of lysozymes considerably more than the unadjuvanted pSCPI. pSCPI + PA and pSCPI + FIA induced substantially higher rates of lysozyme activity compared with pSCPI + AH. Moreover, the combination pSCPI + FIA enhanced more markedly the action of lysozymes than did pSCPI + PA during the three experimental weeks. The highest lysozyme activity was observed after 4 weeks in both adjuvanted pSCPI and unadjuvanted pSCPI, and the treatment with pSCPI + FIA stimulated the highest rates of lysozyme activity.

### 2.4. Specific Antibody Titer Detection

**Figure 4:**
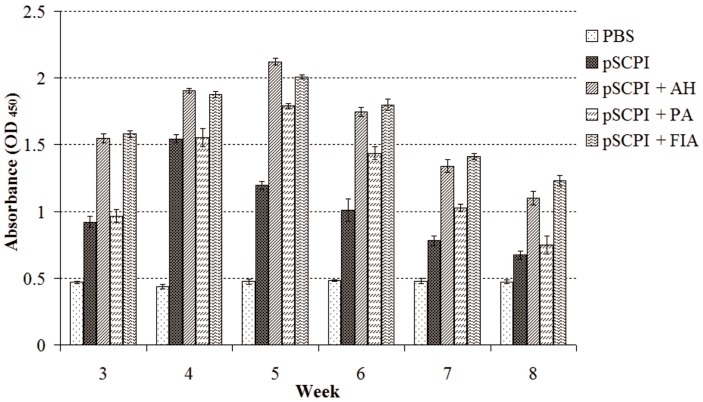
Special antibody titers of vaccinated channel catfish by ELISA. Channel catfish were vaccinated twice, at two-week intervals, with PBS, pSCPI, pSCPI + AH, pSCPI + PA, and pSCPI + FIA. Sera from the three fish were collected and pooled at one-week intervals for 3 to 8 weeks post-vaccination.

The specific antibody titers in sera were evaluated continuously by ELISA from week 3 to 8 post-vaccination (Figure 4). In general, the vaccine group induced higher levels of antibodies than the control group in all 6 weeks. The three different adjuvanted pSCPI treatments also stimulated the production of larger quantities of antibodies compared with the unadjuvanted pSCPI in 6 weeks. pSCPI + AH and pSCPI + FIA induced higher levels of antibodies than pSCPI + PA during the 6 weeks, while the combination pSCPI + AH enhanced the production of antibodies more pronouncedly than pSCPI + FIA only in week 4 and 5. The highest pSCPI antibody titers were observed 4 weeks after the vaccination, while for the adjuvanted pSCPI this effect was established in the fifth week after the administration of the vaccine.

### 2.5. Expression of Immune-Related Genes

**Figure 5:**
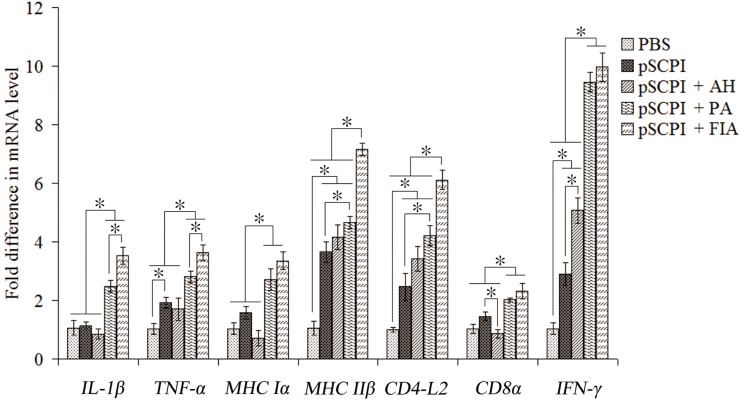
Expression of immune-related genes in vaccinated channel fish determined by qRT-PCR. Channel catfish were vaccinated twice, at two-week intervals, with PBS, pSCPI, pSCPI + AH, pSCPI + PA, and pSCPI + FIA. Total RNA of the five fish from each group was extracted from the head-kidney 48 h post-challenge and used for qRT-PCR. For each gene, the mRNA level of the PBS-vaccinated fish was set as 1. * indicates significant (*p* < 0.05) differences.

The expression of genes encoding interleukin 1β (*IL-1β*), tumor necrosis factor-α (*TNF-α*), major histocompatibility complex (MHC) class I α (*MHC I α*) and II β (*MHC II β*), *CD4-L2*, *CD8 α* and interferon-γ (*IFN-γ*) was examined by qRT-PCR. Compared with the expression in PBS, pSCPI + FIA and pSCPI + PA significantly induced the production of all the investigated genes. The treatment with pSCPI + AH considerably stimulated the induction of *MHC II β*, *CD4-L2*, and *IFN-γ*, while it promoted only a slightly higher production of *TNF-α* and even caused a decline in the levels of *IL-1β*, *MHC I α*, and *CD8 α*. pSCPI alone substantially enhanced the expression of the genes encoding *TNF-α*, *MHC II β*, *CD4-L2*, and *IFN-γ*, whereas it stimulated a moderately higher production of *IL-1β*, *MHC I α*, and *CD8 α* (Figure 5).

## 3. Discussion

The highly conserved streptococcal C5a peptidase (SCP) is an important surface virulence protein and a prime vaccine candidate of Group A streptococci (SCPA) and Group B streptococci (SCPB) in humans and mice [23,24,25]. Since the BLAST (tblastn) analysis indicated that the SCP of *S. iniae* (SCPI) isolated in the hybrid striped bass had equal degrees of similarity to the C5a peptidases of GAS and GBS [7], we concluded that SCPI might also be a vaccine candidate. In our previous work, we found that the entire molecule of SCPI encodes 1122 amino acids, whereas amino acids 1–30 were predicted to be the signal sequence by SignalP 4.1 and amino acids 1100–1117 were predicted as the transmembrane helical region and amino acids 1118–1122 were expected to be located in the intramembrane with TMHMM 2.0. Furthermore, using the conserved domain database of NCBI, it was predicted that SCPI included a Peptidase_S8_S53 superfamily conservative domain (97–319 aa and 437–559 aa, respectively), a PA superfamily conservative domain (338–468 aa), and an Fn3-like superfamily conservative domain (585–695 aa) [26]. Therefore, in the present study, we truncated a part of C5a peptidase to obtain high yields of recombinant proteins; pSCPI, which lacks the signal peptide and contains the conserved domains and most of the B-cell epitopes, was produced. In previous examinations, ELISA was used to detect SCPA [27] or SCPB [25]. Western blotting was used in the present study to confirm the presence of the pSCPI protein. Based on the resulting RPS, pSCPI was demonstrated to possess only moderate immunoprotection efficacy (RPS = 46.43%) and thus could be used for the investigation of its adjuvant effect.

Three different adjuvants were used in this study. Although FCA has been employed as the most effective adjuvant in the largest range of animals, investigation of its role in immunostimulation in fish has been limited [13]. Moreover, in fish, FCA has not always contributed to an enhancement in the immunogenicity or protection [13]. Thus, FIA was used in this study. AH has been utilized in human and most animal vaccines because of its safety [28,29]. PA has also been regarded as a potent adjuvant in veterinary vaccines due to its immunomodulatory and anti-inflammatory effects and low toxicity to experimental animals [30,31,32]. Furthermore, the three adjuvants have been used in aquatic vaccines with proven efficacy. Examples include the successful use of FIA as an adjuvant against GBS infection in tilapia vaccines [10], FIA and AH against *Edwardsiella tarda* in Japanese flounder vaccines [12], AH in olive flounder vaccines against viral hemorrhagic septicemia (VHS) [33], and PA used as an adjuvant in the pentavalent vaccine for turbots [16].

Due to their ectothermic nature and evolutionary status, fish predominantly manifest non-specific immunity, which usually precedes the specific immune response and activation, and its role in the maintenance of homeostasis [34]. Lysozymes have been found in the serum, mucus, and ova of fish, where they play a central role in the innate immune defense mechanisms [35,36]. They are generally effective innate immune factors against bacterial and parasitic infections [37]. Our results suggested that pSCPI induced the production of lysozymes to activate the protective mechanisms against bacterial infections, and the three different adjuvants under investigation enhanced this capacity. FIA manifested the strongest potential among the three adjuvants, which indicated that FIA is a more effective adjuvant than PA and AH.

It is well known that a subunit vaccine has weak immunogenicity. Further, the immune system of fish is slower to react than the immune systems of higher vertebrates [38]. Therefore, the use of adjuvants in the composition of subunit vaccines is essential. After the combinations of recombinant pSCPI with adjuvants were injected intraperitoneally for primary immunization, the adjuvants stimulated the adaptive immune response and enhanced immunological memory [13]. We boosted the immune system with recombinant pSCPI lacking adjuvants to stimulate an optimal immune response [39]. Although pSCPI + AH induced higher antibody levels compared with pSCPI + FIA in weeks 4 and 5, pSCPI + FIA exhibited higher levels of antibodies compared with pSCPI + AH in weeks 6, 7, and 8. The properties of FIA, which releases antigens in a slow and sustained fashion [12], may explain this phenomenon. Therefore, while pSCPI was mixed with FIA, it diffused from the injection site to the visceral organs and persisted for at least 6 weeks.

Ribosomal RNAs constitute between 85% and 90% of the total cellular RNA. As their expression has been shown to be fairly stable under unfavorable conditions, they have been widely used as quantitative references for gene expression [40]. Moreover, rRNAs are superior to the common housekeeping genes (e.g., *ACTB* and *GAPDH*) for normalizing mRNA expression in rats [41], human [42], and mouse [43] tissues and cells. Small *et al*. have reported that *18S rRNA* is the best choice for normalizing real-time PCR data collected from channel catfish tissues experimentally [44]. Therefore, we selected *18S rRNA* to be a housekeeping gene to normalize the real-time PCR data. Aluminum adjuvants are known to induce only a Th2 response [20,21], but the propolis adjuvant [45] and FIA evoked stronger humoral and cellular immunity. The production of *MHC II*, which enables extracellular antigen presentation in conjunction with CD4+ T helper (Th) cells [46], was stimulated significantly in the fish vaccinated with both adjuvanted pSCPI and single pSCPI, which suggested that the Th cells were activated in the vaccinated fish. While *MHC I α* and *CD8 α* are involved in the cellular immunity, and *CD4-L2* participates in humoral immunity, *IFN-γ* exerts an important role in both innate and cellular immune responses [1,47]. On the other hand, *IL-1β* and *TNF-α* are generally related to the inflammatory response and were also expressed higher in the fish vaccinated with both pSCPI + PA and pSCPI + FIA compared to controls. However, while the aluminum adjuvant functioned predominantly in humoral immunity, the expression of *IFN-γ* was enhanced considerably, which might contribute to innate immunity.

RPS is an important parameter to evaluate the effects of vaccines. In the present study, the subunit vaccine pSCPI exhibited moderate immunoprotective efficacy, while the levels of immunoprotection mediated by pSCPI + AH, pSCPI + PA, and pSCPI + FIA were higher and manifested RPS values of 67.86%, 75.00%, and 85.71%, respectively. Thus, FIA manifested the strongest RPS among the three adjuvants, which also indicated that FIA is a more effective adjuvant than PA and AH.

## 4. Materials and Methods

### 4.1. Bacterial Strain, Plasmid and Growth Conditions

The *S. iniae* strain DGX07 was isolated from channel catfish in China and stored in our laboratory (Wenjiang, Sichuan, China) [4]. The pET-32a (+)-pSCPI plasmid was constructed and stored at our laboratory [26]. The *S. iniae* strain DGX07 was cultured in brain-heart infusion medium (BHI, Oxoid, Basingstoke, UK)) at 28 °C, and *Escherichia coli* BL21 (DE3) (Invitrogen, Carlsbad, CA, USA) was cultured in Luria-Bertani (LB) medium at 37 °C. Ampicillin (Sangon Biotech, Shanghai, China) was supplemented at a final concentration of 100 mg/mL.

### 4.2. Animals

To obtain specific antisera, rabbits weighting 2–2.5 kg were acclimated in an animal room for 2 weeks and fed daily with commercial feed. Channel catfish (60.0 ± 5.0 g) were purchased from a fish farm in PuJiang (Sichuan, China) and were acclimatized in a concrete culture tank (1.5 m × 1.0 m × 1.5 m) for 2 weeks before experimental manipulation. The fish were fed a commercial diet daily, and the water was partly replaced every day, maintaining a temperature of 25 °C ± 3 °C. Agglutination test results showed no reaction between the serum and *S. iniae* DGX07. Fish were anaesthetized with MS222 (Sigma, Beijing, China) prior to the experiments, which involved manipulations such as injections and serum collection. All the animal experiments complied with the ethical standards set by the Ethical Committee of the Faculty of Veterinary Medicine (Sichuan Agricultural University, Ya’an, China; Approval No. 2011–028).

### 4.3. Expression and Purification of the pSCPI Protein

*Escherichia coli* BL21 (DE3) strains transformed with pET-32a (+)-p*SCPI* were cultured in LB medium with agitation at 37 °C. When the OD_600nm_ reached 0.6, IPTG (Sigma, St. Louis, MO, USA) was added to a final concentration of 0.1 mM at 37 °C, and the samples in the conical flask were cultured for 4 h. After the induction period, the cultures were centrifuged at 8000× *g* for 10 min at 4 °C and were suspended in 20 mM Tris-HCl buffer. The crude extracts were obtained from sonication using a Sonic Dismembrator Model 500 and examined by 12.5% SDS-PAGE. The inclusion bodies from the insoluble fractions were purified by Ni-NTA-Sefinose Column (Sangon Biotech, Shanghai, China) after dissolution in 8 M urea solutions and passage through 0.22-µm filters. The refolding of the purified proteins was carried out via transition passage from 6 M urea solutions to PBS (phosphate buffered saline solution) via gradient dialysis at 4 °C, and then analyzed by 12.5% SDS-PAGE. The quantity was determined using a BIO-RAD Smart Spec Plus (Bio-Rad Laboratories, Inc., Hercules, CA, USA.) according to the manufacturer’s instructions. Purified proteins were stored at −20 °C.

### 4.4. Preparation of Specific Antisera

New Zealand white rabbits were divided into an experimental group and a control group. The purified pSCPI (1.2 mg/mL) was used as the antigen and mixed with an equal volume of FCA (Sigma, St. Louis, MO, USA). This mixture was injected into the rabbits in the experimental group, followed by three booster shots of pSCPI + FIA by subcutaneous injection at one-week intervals. The rabbits immunized with PBS were utilized as a control. Three days after the final injection with pSCPI + FIA, blood was collected from the rabbits in both groups, which was subsequently clarified, and centrifuged at 3000× *g* for 15 min. The serum was prepared and stored at −20 °C. Immunoglobulin G (IgG) was purified by the ammonium sulfate precipitation method [48].

### 4.5. Western Blotting

The purified proteins were separated by 12% SDS-PAGE and transferred to a PVDF membrane electrically at 150 V for 4 h. After pre-blocking with TBST (containing 3% BSA) for 1 h at room temperature, the membrane was incubated with rabbit anti-pSCPI/negative sera diluted 1:100 in TBST (containing 0.5% BSA) for 12 h at 4 °C. After washing with TBS, the membrane was incubated with goat-anti-rabbit IgG (H + L)-HRP (Sigma, St. Louis, MO, USA) diluted 1:5000 in TBST (containing 3% BSA) for 1 h. The reaction was visualized using DAB (Sigma) for 5 to 15 min, and was terminated by rinsing with distilled water [49].

### 4.6. Preparation of the Adjuvant and Vaccine

The purified pSCPI (1.2 mg/mL) admixed with propolis adjuvants (pSCPI + PA) was prepared based on the following procedure [50]: 10 g propolis (bought at the JiangShi bee garden, Ya’an, China) and 95% ethanol were mixed in a ratio of 1:4 (*w*/*v*). The mixtures were incubated at 25 °C for 24 h with stirring and were sterilized by passing through 0.22-µm filters. Finally, the mixed solutions were resuspended in PBS to 30 mg/mL. To obtain pSCPI + PA, the purified recombinant pSCPI was mixed in equal volumes with the propolis adjuvants.

The purified pSCPI adjuvanted with aluminum hydroxide (pSCPI + AH) was prepared as follows [12]: 5% Al_2_(SO_4_)_3_ and 5% NaOH were filter-sterilized, as described above. The solutions were incubated at 60 °C for 30 min. Five volumes of 5% Al_2_(SO_4_)_3_ and two volumes of 5% NaOH were mixed with stirring, followed by centrifugation at 10,000× *g* for 5 min. After washing twice with sterile PBS, the mixtures were resuspended in PBS to 0.2 mg/mL. The purified recombinant pSCPI was mixed with an equal volume of aluminum hydroxide adjuvant to obtain pSCPI + AH.

The purified pSCPI (1.2 mg/mL) adjuvanted with FIA (pSCPI + FIA) was prepared by mixing pSCPI and FIA (Sigma, St. Louis, MO, USA) in equal volumes until a stable emulsion (a homogeneous white mixture) was formed [12].

All the adjuvanted vaccines above were stored at 4 °C until use (no more than three days).

### 4.7. Fish immunization and Challenge

Three hundred channel catfish were divided into five groups of 60 fish each: four vaccine-treated groups and one control group. The individual fish in the four vaccine groups, namely, pSCPI + FIA, pSCPI + AH, pSCPI + PA, and pSCPI, were injected intraperitoneally with 200 µL pSCPI + FIA, pSCPI + AH, pSCPI + PA, and pSCPI, respectively. The control (PBS) group received an identical dose of PBS. 2 weeks post-vaccination, the fish in the four vaccine groups were boosted with 200 µL purified pSCPI.

On the 28th day after booster vaccination, 20 fish were randomly selected from each treatment group, and challenged with 200 µL of the *S. iniae* strain DGX07 at 6 × 10^7^ CFU/mL [4] by intraperitoneal injection. A period of 14 days after the challenge, the mortality was recorded daily. RPS was calculated according to the formula: RPS = [1− (%mortality of vaccinated fish/% mortality of control fish)] × 100% [51].

The 40 remaining fish were sampled at week 3 to 8 during the trial. Serum samples from five individuals in each group were collected for assessment of lysozyme activity and ELISA. Individual sera from each group were pooled to conduct the above-mentioned assays, which were repeated three times for all samples. Head-kidney samples from five fish were taken for qRT-PCR 48 h post-challenge.

### 4.8. Detection of Lysozyme Activity

The lysozyme activity was assessed using the previously described method [52,53]. Briefly, a turbidimetric assessment utilizing lyophilized *Micrococcus lysodeikticus* cells (Sigma) was performed. *M. lysodeikticus* (150 µL) with a concentration of 0.2 mg/mL (in 0.02 M sodium citrate buffer, pH = 5.5) was added to 15 µL sera in a 96-well U-bottom microtiter plate. The initial optical density (OD) was detected at 450 nm instantly after adding *M. lysodeikticus*, and then, the final OD was measured after incubation for 1 h at 37 °C. Lyophilized hen egg-white lysozyme (Sigma) was used to develop a standard curve. The lysozyme levels were expressed as units·mL^−1^, with a decrease in absorbance of 0.001 min^−1^ representing one unit.

### 4.9. ELISA

Specific antibody titers for pSCPI were determined by ELISA [54]. In brief, the pSCPI was diluted to a concentration of 500 μg/mL in a carbonate buffer (pH = 9.6). Each well of the 96-well plate was covered with 100 µL diluted pSCPI overnight at 4 °C followed by washing with PBST (0.1% Tween-20 in PBS) and blocking with 3% bovine serum albumin (BSA) in PBST for 2 h at 37 °C. Serial 2-fold dilutions of sera were added to the wells in triplicate and subsequently incubated for 2 h at 37 °C. Rabbit anti-channel catfish IgM antibody (produced in our laboratory) (1:6400) and goat-anti-rabbit IgG (H + L)-HRP (1:2000) were used as the secondary and tertiary antibodies, respectively. The TMB kit (Tiangen, Beijing, China) was used for color development. The plates were read with a microplate reader (Bio-Rad, Hercules, CA, USA) at 450 nm.

### 4.10. Quantitative Real-Time Reverse Transcription-PCR (qRT-PCR) Analysis of the Expression of Immune-Related Genes

Head-kidney samples were obtained from the vaccinated fish at 48 h post-challenge. Total RNA was extracted from the kidney by the RNAprep Tissue/Bacteria Kit (Takara, Dalian, China), following the manufacturer’s instructions. The cDNA was synthesized using a RevertAid First Strand cDNA Synthesis kit (Takara, Dalian, China) according to the manufacture’s guidelines. The qRT-PCR was performed with the ABI PlusOne System (Applied Biosystems, Foster City, CA, USA) with primers (All were listed in Table 1) using the SYBR ExScript qRT-PCR Kit (Takara, Dalian, China), as described previously [55]. The housekeeping gene 18S rRNA was regarded as the control for cDNA quantity and quality. All the listed primers used in this assay spanned exons of the respective genes. A melting curve analysis of the amplification products was routinely performed at the end of each PCR cycle to confirm that the single PCR product was amplified and detected. For each gene, a standard curve was generated to estimate the amplification efficiency. Negative controls with no template were always included in the reactions. Finally, the expression level of immune-related genes was analyzed via the comparative threshold cycle method (2^−ΔΔ*C*t^ method). All data were presented in terms of relative mRNA and expressed as means ± SE.

**Table 1:** Primers used in this study.

Primer	Sequences (5′→3′)	Target Gene	Product Size (bp)	GenBank
18S F	GGACACGGAAAGGATTGACAGA	*18S rRNA*	121	AF021880.1
18S R	GAGGAGTCTCGTTCGTTATCGG		174	DQ160229.1
IL-1β1 F	GCCATGTTGCTAATGTTGTAATCG	*IL-1* *β*	146	U77598.1
IL-1β1 R	TGTCTTGCAGGCTGTAACTCTTG		129	AJ417565.2
MHC II F	CGGGAAGGAGATTAAAGGAGGT	*MHC II* *β*	100	AF053547.1
MHC II R	GTTTGGTGAAGCTGGCGTGT		122	NM_001200227.1
TNF α F	CGCACAACAAACCAGACGAGAC	*TNF-* *α*	122	GQ179649.1
TNF α R	ACCACTGCATAGATACGCTCGAA		100	NM_001200217.1
MHC I F	GGTATCATCGTTGGTGTAGCCG	*MHC I* *α*	121	AF021880.1
MHC I R	GGACAGGTTTGAAGCCAGAGTT		174	DQ160229.1
CD4 F	GCAGGGCACGGATAGATGGA	*CD4-L2*	146	U77598.1
CD4 R	TGGGTTCGCAGAGGCTGATAC		129	AJ417565.2
CD8 F	CCGACAGTGCCTACGACTAAAGC	*CD8* *α*	100	AF053547.1
CD8 R	CCAGCAGCCAAAGGAATGAAG		122	NM_001200227.1
IFN F	TGCACGAAGTGAAAGACCAAA	*IFN-γ*	122	GQ179649.1
IFN R	TTAAGGTCCAGCAGCTCAGTGA		100	NM_001200217.1

### 4.11. Statistical Analysis

All data were analyzed by one-way analysis of variance and Duncan’s test using the SPSS 19.0 package (SPSS Inc., Chicago, IL, USA). All results were shown as mean ± SE, and the differences were considered statistically non-significant and significant when *p* > 0.05 and *p* < 0.05, respectively. * *p* < 0.05.

## 5. Conclusions

In conclusion, the subunit vaccine pSCPI possesses immunogenicity and exerts moderate immune protection against *S. iniae* infection in channel catfish. Our findings *in* *vitro* revealed that the three different adjuvants (AH, PA, and FIA) triggered an immune response and improved the immune protection of pSCPI. Of the three adjuvants, FIA exhibited the maximum immunostimulation, followed by PA and AH. Therefore, compared to the other two adjuvants used in our study, FIA is a more promising candidate adjuvant, in combination with pSCPI, against *S. iniae* infections in channel catfish.
